# Crystal structure of 1-(2-chloro­acet­yl)-3,3-dimethyl-2,6-di-*p*-tolyl­piperidin-4-one

**DOI:** 10.1107/S2056989015002613

**Published:** 2015-02-13

**Authors:** S. Jothivel, Jibon Kotoky, S. Kabilan

**Affiliations:** a Drug Discovery Lab., Department of Chemistry, Annamalai University, Annamalai Nagar, Tamil Nadu 608 002, India; b Division of Life Sciences, Central Instrumentation Facility, Institute of Advanced Study in Science & Technology (IASST), Guwahati 781 035, Assam, India

**Keywords:** crystal structure, piperidones, piperidin-4-one, *p*-tol­yl

## Abstract

In the title compound, C_23_H_26_ClNO_2_, the piperidin-4-one ring adopts a distorted boat conformation. The two *p*-tolyl rings are nearly normal to each other, making a dihedral angle of 83.33 (10)°. They are inclined to the mean plane of the piperidine ring by 73.2 (1) and 87.22 (9)°. In the crystal, there are no significant inter­molecular inter­actions present.

## Related literature   

For some biological properties of piperidones, see: Dimmock *et al.* (2001 ▸); Perumal *et al.* (2001 ▸). For the synthesis of the title compound, see: Aridoss *et al.* (2007 ▸). For further literature on piperidones and the crystal structures of similar compounds, see: Parthiban *et al.* (2009 ▸); Ravindran *et al.* (1991 ▸); Krishnakumar & Krishnapillay (1996 ▸).
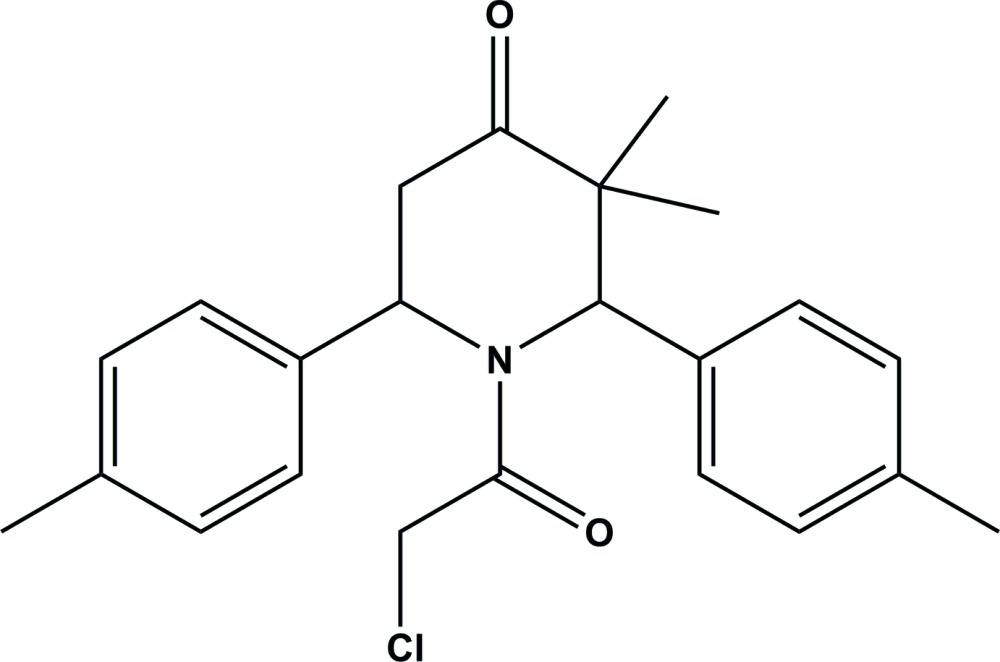


## Experimental   

### Crystal data  

C_23_H_26_ClNO_2_
*M* *_r_* = 383.90
Monoclinic, 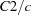

*a* = 18.7923 (6) Å
*b* = 18.8289 (5) Å
*c* = 11.6689 (3) Å
β = 93.162 (2)°
*V* = 4122.6 (2) Å^3^
*Z* = 8
Mo *K*α radiation
μ = 0.20 mm^−1^
*T* = 296 K
0.35 × 0.30 × 0.25 mm

### Data collection  

Bruker Kappa APEXII CCD diffractometer
Absorption correction: multi-scan (*SADABS*; Bruker, 2004 ▸) *T*_min_ = 0.931, *T*_max_ = 0.959
29055 measured reflections
3989 independent reflections
3097 reflections with *I* > 2σ(*I*)
*R*_int_ = 0.028

### Refinement  

*R*[*F* ^2^ > 2σ(*F* ^2^)] = 0.041
*wR*(*F* ^2^) = 0.124
*S* = 1.03
3989 reflections
244 parameters
H-atom parameters constrained
Δρ_max_ = 0.30 e Å^−3^
Δρ_min_ = −0.22 e Å^−3^

### 

Data collection: *APEX2* (Bruker, 2004 ▸); cell refinement: *APEX2* and *SAINT* (Bruker, 2004 ▸); data reduction: *SAINT* and *XPREP* (Bruker, 2004 ▸); program(s) used to solve structure: *SIR92* (Altomare *et al.*, 1993 ▸); program(s) used to refine structure: *SHELXL97* (Sheldrick, 2008 ▸); molecular graphics: *ORTEP-3 for Windows* (Farrugia, 2012 ▸); software used to prepare material for publication: *SHELXL97* and *PLATON* (Spek, 2009 ▸).

## Supplementary Material

Crystal structure: contains datablock(s) I, publication_text. DOI: 10.1107/S2056989015002613/su5059sup1.cif

Structure factors: contains datablock(s) I. DOI: 10.1107/S2056989015002613/su5059Isup2.hkl

Supporting information file. DOI: 10.1107/S2056989015002613/su5059Isup3.cml

**Fig. 1.**
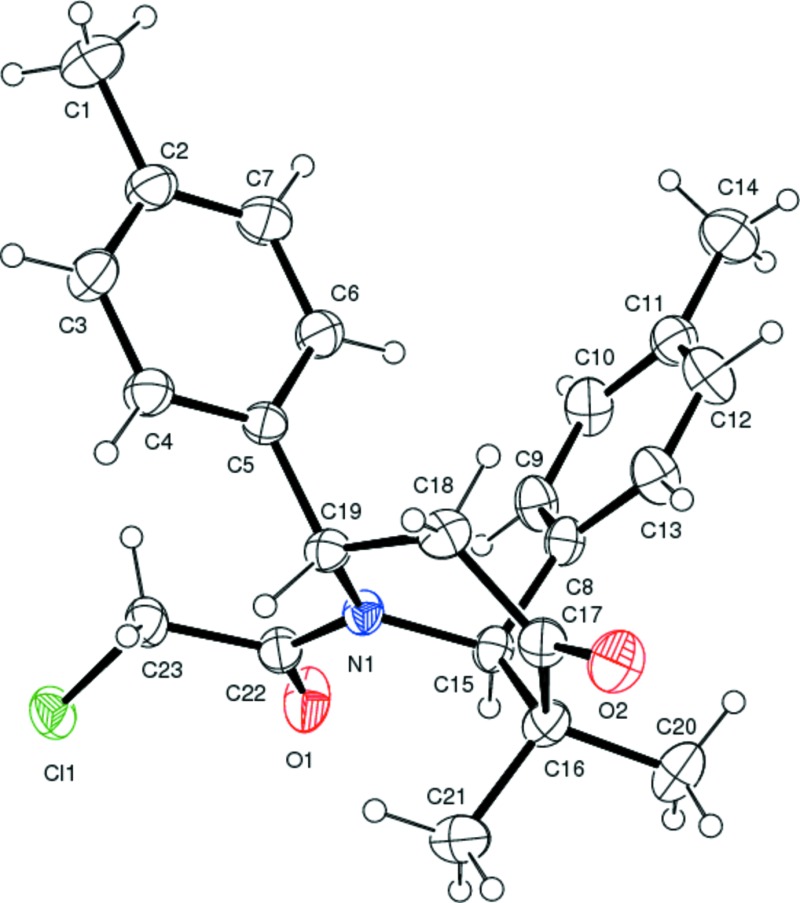
The molecular structure of the title compound, with atom labelling. Displacement ellipsoids are drawn at the 30% probability level.

. DOI: 10.1107/S2056989015002613/su5059fig1.tif
The mol­ecular structure of the title compound, with atom labelling. Displacement ellipsoids are drawn at the 30% probability level.

CCDC reference: 1030980

Additional supporting information:  crystallographic information; 3D view; checkCIF report

## supplementary crystallographic information

### S1. Comment

Piperidones are an important group of heterocyclic compounds in the field of medicinal chemistry due to their biological activities, including cytotoxic properties (Dimmock *et al.*, 2001). They were also reported to possess analgesic, anti-inflammatory, central nervous system (*CNS*), local anaesthetic, anticancer and antimicrobial activities (Perumal *et al.*, 2001). The present investigation was undertaken to establish the structure, conformation of the heterocyclic ring and orientation of the 4-tolyl groups in the title compound.

The molecular structure of the title compound is illustrated in Fig. 1. The sum of the bond angles around atom N1 is 359.39° indicating sp^2^ hybridization. The N1—C22 [1.349 (2) Å] and C22—O1[1.218 (2) Å] bond distances indicate electron delocalization. The six membered piperidine ring (N1/C15-C19) adopts a distorted boat conformation. The two *p*-tolyl rings are nearly orthogonal to each other with a dihedral angle of 83.33 (10)°. The methyl substituents are oriented equatorially [N1—C15—C16—C20 = 175 (16)°] and axially [N1—C15—C16—C21 = 56.52 (19)°] at the C3 position. The two *p*-tolyl (C2-C7 and C8-C13) are inclined to the mean plane of the piperidine ring by 73.2 (1) and 87.22 (9) °, respectively.

In the crystal, there are no significant intermolecular interactions present.

### S2. Experimental

The title compound was synthesized according to a published procedure (Aridoss *et al.*, 2007). To a well stirred solution of 3, 3-dimethyl-2, 6-di-*p*-tolyl piperidin-4-one (5 mmol), and triethylamine (5 mmol) in 20 ml of benzene, dichloroacetylchloride (5 mmol) in 20 ml of benzene was added drop wise through the additional funnel over ca. 30 min. Stirring was continued with mild heating using a magnetic stirrer for 7 h. The progress of the reaction was monitored by TLC. After completion of reaction, the mixture was poured into water and extracted with ether. The collected ether extracts were then washed well with 3% sodium bicarbonate solution and dried over anhydrous Na_2_SO_4_. The pasty mass obtained was purified by crystallization from distilled ethanol giving the compound in pure form as colourless block-like crystals.

### S3. Refinement

H atoms were positioned geometrically and refined using a riding model: C—H = 0.93–0.98 Å with *U*_iso_(H) = 1.5U_eq_(C) for methyl H atoms and = 1.2U_eq_(C) for other H atoms.

### Crystal data

**Table:**

C_23_H_26_ClNO_2_	*F*(000) = 1632
*M**_r_* = 383.90	*D*_x_ = 1.237 Mg m^−^^3^
Monoclinic, *C*2/*c*	Mo *K*α radiation, λ = 0.71073 Å
Hall symbol: -C 2yc	Cell parameters from 8523 reflections
*a* = 18.7923 (6) Å	θ = 2.3–25.5°
*b* = 18.8289 (5) Å	µ = 0.20 mm^−^^1^
*c* = 11.6689 (3) Å	*T* = 296 K
β = 93.162 (2)°	Block, colourless
*V* = 4122.6 (2) Å^3^	0.35 × 0.30 × 0.25 mm
*Z* = 8	

### Data collection

**Table:**

Bruker Kappa APEXII CCD diffractometer	3989 independent reflections
Radiation source: fine-focus sealed tube	3097 reflections with *I* > 2σ(*I*)
Graphite monochromator	*R*_int_ = 0.028
ω and φ scan	θ_max_ = 25.8°, θ_min_ = 2.2°
Absorption correction: multi-scan (*SADABS*; Bruker, 2004)	*h* = −23→22
*T*_min_ = 0.931, *T*_max_ = 0.959	*k* = −23→23
29055 measured reflections	*l* = −14→14

### Refinement

**Table:**

Refinement on *F*^2^	Primary atom site location: structure-invariant direct methods
Least-squares matrix: full	Secondary atom site location: difference Fourier map
*R*[*F*^2^ > 2σ(*F*^2^)] = 0.041	Hydrogen site location: inferred from neighbouring sites
*wR*(*F*^2^) = 0.124	H-atom parameters constrained
*S* = 1.03	*w* = 1/[σ^2^(*F*_o_^2^) + (0.0577*P*)^2^ + 3.5027*P*] where *P* = (*F*_o_^2^ + 2*F*_c_^2^)/3
3989 reflections	(Δ/σ)_max_ < 0.001
244 parameters	Δρ_max_ = 0.30 e Å^−^^3^
0 restraints	Δρ_min_ = −0.22 e Å^−^^3^

### Special details

**Table:**

Geometry. All e.s.d.'s (except the e.s.d. in the dihedral angle between two l.s. planes) are estimated using the full covariance matrix. The cell e.s.d.'s are taken into account individually in the estimation of e.s.d.'s in distances, angles and torsion angles; correlations between e.s.d.'s in cell parameters are only used when they are defined by crystal symmetry. An approximate (isotropic) treatment of cell e.s.d.'s is used for estimating e.s.d.'s involving l.s. planes.
Refinement. Refinement of *F*^2^ against ALL reflections. The weighted *R*-factor *wR* and goodness of fit *S* are based on *F*^2^, conventional *R*-factors *R* are based on *F*, with *F* set to zero for negative *F*^2^. The threshold expression of *F*^2^ > σ(*F*^2^) is used only for calculating *R*-factors(gt) *etc*. and is not relevant to the choice of reflections for refinement. *R*-factors based on *F*^2^ are statistically about twice as large as those based on *F*, and *R*- factors based on ALL data will be even larger.

### Fractional atomic coordinates and isotropic or equivalent isotropic displacement parameters (Å^2^)

**Table:**

	*x*	*y*	*z*	*U*_iso_*/*U*_eq_	
C1	0.99934 (14)	0.15212 (14)	0.4159 (3)	0.0785 (8)	
H1A	0.9762	0.1118	0.3802	0.118*	
H1B	1.0097	0.1423	0.4959	0.118*	
H1C	1.0429	0.1616	0.3795	0.118*	
C2	0.95102 (11)	0.21596 (11)	0.40374 (19)	0.0516 (5)	
C3	0.97183 (11)	0.28078 (11)	0.44787 (18)	0.0532 (5)	
H3	1.0169	0.2854	0.4841	0.064*	
C4	0.92751 (10)	0.33922 (10)	0.43974 (17)	0.0466 (5)	
H4	0.9431	0.3823	0.4711	0.056*	
C5	0.86037 (9)	0.33477 (9)	0.38582 (15)	0.0372 (4)	
C6	0.83961 (11)	0.26980 (10)	0.34030 (19)	0.0519 (5)	
H6	0.7949	0.2652	0.3029	0.062*	
C7	0.88421 (12)	0.21166 (11)	0.3495 (2)	0.0603 (6)	
H7	0.8688	0.1685	0.3183	0.072*	
C8	0.67209 (9)	0.34288 (10)	0.22260 (15)	0.0399 (4)	
C9	0.68688 (10)	0.30852 (12)	0.12166 (17)	0.0501 (5)	
H9	0.7118	0.3326	0.0669	0.060*	
C10	0.66551 (12)	0.23935 (12)	0.1004 (2)	0.0580 (6)	
H10	0.6770	0.2177	0.0322	0.070*	
C11	0.62763 (11)	0.20178 (11)	0.1777 (2)	0.0540 (5)	
C12	0.61085 (12)	0.23649 (12)	0.27655 (19)	0.0607 (6)	
H12	0.5842	0.2129	0.3296	0.073*	
C13	0.63251 (11)	0.30541 (12)	0.29906 (18)	0.0529 (5)	
H13	0.6203	0.3271	0.3668	0.064*	
C14	0.60485 (15)	0.12615 (13)	0.1559 (3)	0.0794 (8)	
H14A	0.5792	0.1093	0.2194	0.119*	
H14B	0.6462	0.0970	0.1475	0.119*	
H14C	0.5747	0.1239	0.0869	0.119*	
C15	0.70211 (9)	0.41716 (10)	0.24177 (16)	0.0388 (4)	
H15	0.6940	0.4422	0.1686	0.047*	
C16	0.66973 (10)	0.46453 (10)	0.33298 (16)	0.0433 (4)	
C17	0.68764 (10)	0.43549 (10)	0.45226 (16)	0.0426 (4)	
C18	0.75270 (10)	0.38867 (10)	0.46625 (16)	0.0449 (4)	
H18A	0.7370	0.3396	0.4611	0.054*	
H18B	0.7738	0.3958	0.5432	0.054*	
C19	0.81131 (9)	0.39888 (9)	0.38170 (15)	0.0369 (4)	
H19	0.8397	0.4402	0.4071	0.044*	
C20	0.58947 (11)	0.47386 (14)	0.3074 (2)	0.0619 (6)	
H20A	0.5810	0.4923	0.2312	0.093*	
H20B	0.5710	0.5064	0.3618	0.093*	
H20C	0.5661	0.4288	0.3134	0.093*	
C21	0.70482 (13)	0.53857 (11)	0.33111 (19)	0.0565 (5)	
H21A	0.6953	0.5600	0.2572	0.085*	
H21B	0.7554	0.5339	0.3459	0.085*	
H21C	0.6856	0.5680	0.3891	0.085*	
C22	0.82141 (10)	0.43472 (10)	0.17806 (16)	0.0419 (4)	
C23	0.90131 (10)	0.43746 (11)	0.20559 (18)	0.0494 (5)	
H23A	0.9206	0.3897	0.2052	0.059*	
H23B	0.9106	0.4570	0.2819	0.059*	
N1	0.78074 (7)	0.41404 (8)	0.26376 (12)	0.0364 (3)	
O1	0.79731 (8)	0.44923 (10)	0.08186 (12)	0.0640 (4)	
O2	0.65195 (8)	0.44814 (8)	0.53268 (12)	0.0578 (4)	
Cl1	0.94435 (3)	0.48999 (3)	0.10507 (5)	0.05769 (18)	

### Atomic displacement parameters (Å^2^)

**Table:**

	*U*^11^	*U*^22^	*U*^33^	*U*^12^	*U*^13^	*U*^23^
C1	0.0700 (16)	0.0599 (15)	0.106 (2)	0.0246 (12)	0.0088 (15)	0.0049 (14)
C2	0.0500 (12)	0.0459 (11)	0.0597 (13)	0.0105 (9)	0.0113 (10)	0.0060 (10)
C3	0.0437 (11)	0.0577 (13)	0.0573 (13)	0.0083 (9)	−0.0054 (9)	0.0013 (10)
C4	0.0461 (10)	0.0427 (11)	0.0499 (11)	0.0013 (8)	−0.0072 (8)	−0.0017 (9)
C5	0.0391 (9)	0.0377 (9)	0.0348 (9)	0.0004 (7)	0.0019 (7)	0.0050 (7)
C6	0.0448 (11)	0.0429 (11)	0.0669 (14)	−0.0011 (9)	−0.0084 (9)	−0.0048 (10)
C7	0.0619 (14)	0.0384 (11)	0.0804 (16)	0.0004 (10)	0.0013 (12)	−0.0094 (10)
C8	0.0325 (9)	0.0502 (11)	0.0366 (9)	−0.0013 (8)	−0.0010 (7)	−0.0024 (8)
C9	0.0458 (11)	0.0631 (13)	0.0421 (11)	−0.0107 (9)	0.0082 (8)	−0.0081 (9)
C10	0.0530 (12)	0.0657 (14)	0.0558 (13)	−0.0064 (10)	0.0087 (10)	−0.0222 (11)
C11	0.0475 (11)	0.0507 (12)	0.0632 (14)	−0.0044 (9)	−0.0033 (10)	−0.0054 (10)
C12	0.0657 (14)	0.0617 (14)	0.0556 (13)	−0.0186 (11)	0.0106 (11)	0.0026 (11)
C13	0.0562 (12)	0.0604 (13)	0.0432 (11)	−0.0132 (10)	0.0111 (9)	−0.0082 (9)
C14	0.0800 (17)	0.0545 (14)	0.103 (2)	−0.0098 (13)	−0.0022 (15)	−0.0091 (14)
C15	0.0326 (9)	0.0460 (10)	0.0373 (9)	0.0011 (7)	−0.0022 (7)	0.0009 (8)
C16	0.0405 (10)	0.0446 (10)	0.0442 (11)	0.0056 (8)	−0.0030 (8)	−0.0038 (8)
C17	0.0431 (10)	0.0423 (10)	0.0427 (10)	−0.0013 (8)	0.0033 (8)	−0.0077 (8)
C18	0.0504 (11)	0.0487 (11)	0.0355 (10)	0.0053 (9)	0.0011 (8)	0.0014 (8)
C19	0.0391 (9)	0.0370 (9)	0.0339 (9)	0.0012 (7)	−0.0044 (7)	0.0012 (7)
C20	0.0449 (12)	0.0752 (15)	0.0650 (14)	0.0169 (11)	−0.0015 (10)	−0.0091 (12)
C21	0.0664 (14)	0.0447 (11)	0.0574 (13)	0.0061 (10)	−0.0057 (11)	−0.0001 (10)
C22	0.0396 (10)	0.0468 (10)	0.0392 (10)	−0.0015 (8)	−0.0002 (8)	0.0064 (8)
C23	0.0396 (10)	0.0571 (12)	0.0516 (12)	−0.0056 (9)	0.0025 (8)	0.0161 (10)
N1	0.0324 (7)	0.0433 (8)	0.0331 (8)	−0.0007 (6)	−0.0022 (6)	0.0033 (6)
O1	0.0448 (8)	0.1056 (13)	0.0410 (8)	−0.0026 (8)	−0.0019 (6)	0.0212 (8)
O2	0.0571 (9)	0.0680 (10)	0.0495 (8)	0.0061 (7)	0.0138 (7)	−0.0085 (7)
Cl1	0.0489 (3)	0.0632 (3)	0.0617 (3)	−0.0087 (2)	0.0092 (2)	0.0175 (3)

### Geometric parameters (Å, º)

**Table:**

C1—C2	1.508 (3)	C14—H14B	0.9600
C1—H1A	0.9600	C14—H14C	0.9600
C1—H1B	0.9600	C15—N1	1.487 (2)
C1—H1C	0.9600	C15—C16	1.540 (3)
C2—C3	1.373 (3)	C15—H15	0.9800
C2—C7	1.377 (3)	C16—C17	1.516 (3)
C3—C4	1.380 (3)	C16—C20	1.531 (3)
C3—H3	0.9300	C16—C21	1.543 (3)
C4—C5	1.381 (3)	C17—O2	1.207 (2)
C4—H4	0.9300	C17—C18	1.509 (3)
C5—C6	1.381 (3)	C18—C19	1.531 (3)
C5—C19	1.518 (2)	C18—H18A	0.9700
C6—C7	1.379 (3)	C18—H18B	0.9700
C6—H6	0.9300	C19—N1	1.489 (2)
C7—H7	0.9300	C19—H19	0.9800
C8—C9	1.385 (3)	C20—H20A	0.9600
C8—C13	1.386 (3)	C20—H20B	0.9600
C8—C15	1.520 (3)	C20—H20C	0.9600
C9—C10	1.381 (3)	C21—H21A	0.9600
C9—H9	0.9300	C21—H21B	0.9600
C10—C11	1.376 (3)	C21—H21C	0.9600
C10—H10	0.9300	C22—O1	1.218 (2)
C11—C12	1.377 (3)	C22—N1	1.349 (2)
C11—C14	1.505 (3)	C22—C23	1.519 (3)
C12—C13	1.381 (3)	C23—Cl1	1.7644 (19)
C12—H12	0.9300	C23—H23A	0.9700
C13—H13	0.9300	C23—H23B	0.9700
C14—H14A	0.9600		
			
C2—C1—H1A	109.5	C8—C15—C16	118.50 (15)
C2—C1—H1B	109.5	N1—C15—H15	106.0
H1A—C1—H1B	109.5	C8—C15—H15	106.0
C2—C1—H1C	109.5	C16—C15—H15	106.0
H1A—C1—H1C	109.5	C17—C16—C20	112.73 (17)
H1B—C1—H1C	109.5	C17—C16—C15	110.46 (15)
C3—C2—C7	117.28 (18)	C20—C16—C15	110.77 (16)
C3—C2—C1	121.0 (2)	C17—C16—C21	105.40 (15)
C7—C2—C1	121.7 (2)	C20—C16—C21	108.11 (17)
C2—C3—C4	121.64 (19)	C15—C16—C21	109.16 (16)
C2—C3—H3	119.2	O2—C17—C18	120.85 (18)
C4—C3—H3	119.2	O2—C17—C16	122.55 (18)
C3—C4—C5	121.03 (18)	C18—C17—C16	116.60 (16)
C3—C4—H4	119.5	C17—C18—C19	117.67 (16)
C5—C4—H4	119.5	C17—C18—H18A	107.9
C4—C5—C6	117.42 (17)	C19—C18—H18A	107.9
C4—C5—C19	120.27 (16)	C17—C18—H18B	107.9
C6—C5—C19	122.27 (16)	C19—C18—H18B	107.9
C7—C6—C5	121.02 (19)	H18A—C18—H18B	107.2
C7—C6—H6	119.5	N1—C19—C5	112.60 (14)
C5—C6—H6	119.5	N1—C19—C18	111.41 (14)
C2—C7—C6	121.6 (2)	C5—C19—C18	109.69 (14)
C2—C7—H7	119.2	N1—C19—H19	107.6
C6—C7—H7	119.2	C5—C19—H19	107.6
C9—C8—C13	116.72 (18)	C18—C19—H19	107.6
C9—C8—C15	117.68 (17)	C16—C20—H20A	109.5
C13—C8—C15	125.58 (17)	C16—C20—H20B	109.5
C10—C9—C8	121.54 (19)	H20A—C20—H20B	109.5
C10—C9—H9	119.2	C16—C20—H20C	109.5
C8—C9—H9	119.2	H20A—C20—H20C	109.5
C11—C10—C9	121.5 (2)	H20B—C20—H20C	109.5
C11—C10—H10	119.2	C16—C21—H21A	109.5
C9—C10—H10	119.2	C16—C21—H21B	109.5
C10—C11—C12	117.13 (19)	H21A—C21—H21B	109.5
C10—C11—C14	121.9 (2)	C16—C21—H21C	109.5
C12—C11—C14	121.0 (2)	H21A—C21—H21C	109.5
C11—C12—C13	121.8 (2)	H21B—C21—H21C	109.5
C11—C12—H12	119.1	O1—C22—N1	123.42 (17)
C13—C12—H12	119.1	O1—C22—C23	120.02 (17)
C12—C13—C8	121.27 (19)	N1—C22—C23	116.54 (15)
C12—C13—H13	119.4	C22—C23—Cl1	111.25 (13)
C8—C13—H13	119.4	C22—C23—H23A	109.4
C11—C14—H14A	109.5	Cl1—C23—H23A	109.4
C11—C14—H14B	109.5	C22—C23—H23B	109.4
H14A—C14—H14B	109.5	Cl1—C23—H23B	109.4
C11—C14—H14C	109.5	H23A—C23—H23B	108.0
H14A—C14—H14C	109.5	C22—N1—C15	117.32 (14)
H14B—C14—H14C	109.5	C22—N1—C19	122.34 (14)
N1—C15—C8	110.26 (14)	C15—N1—C19	119.73 (14)
N1—C15—C16	109.30 (14)		
			
C7—C2—C3—C4	0.8 (3)	C8—C15—C16—C21	−176.06 (15)
C1—C2—C3—C4	−178.4 (2)	C20—C16—C17—O2	−31.5 (3)
C2—C3—C4—C5	−0.5 (3)	C15—C16—C17—O2	−156.03 (18)
C3—C4—C5—C6	−0.2 (3)	C21—C16—C17—O2	86.2 (2)
C3—C4—C5—C19	177.66 (18)	C20—C16—C17—C18	147.89 (18)
C4—C5—C6—C7	0.6 (3)	C15—C16—C17—C18	23.4 (2)
C19—C5—C6—C7	−177.3 (2)	C21—C16—C17—C18	−94.40 (19)
C3—C2—C7—C6	−0.4 (3)	O2—C17—C18—C19	−154.92 (18)
C1—C2—C7—C6	178.8 (2)	C16—C17—C18—C19	25.7 (2)
C5—C6—C7—C2	−0.2 (4)	C4—C5—C19—N1	129.92 (18)
C13—C8—C9—C10	−2.4 (3)	C6—C5—C19—N1	−52.3 (2)
C15—C8—C9—C10	175.93 (18)	C4—C5—C19—C18	−105.40 (19)
C8—C9—C10—C11	1.0 (3)	C6—C5—C19—C18	72.4 (2)
C9—C10—C11—C12	1.0 (3)	C17—C18—C19—N1	−37.9 (2)
C9—C10—C11—C14	−179.3 (2)	C17—C18—C19—C5	−163.28 (15)
C10—C11—C12—C13	−1.6 (3)	O1—C22—C23—Cl1	19.9 (3)
C14—C11—C12—C13	178.7 (2)	N1—C22—C23—Cl1	−161.74 (15)
C11—C12—C13—C8	0.2 (4)	O1—C22—N1—C15	−7.0 (3)
C9—C8—C13—C12	1.8 (3)	C23—C22—N1—C15	174.71 (16)
C15—C8—C13—C12	−176.37 (19)	O1—C22—N1—C19	−177.95 (18)
C9—C8—C15—N1	−70.5 (2)	C23—C22—N1—C19	3.7 (3)
C13—C8—C15—N1	107.6 (2)	C8—C15—N1—C22	105.19 (18)
C9—C8—C15—C16	162.48 (17)	C16—C15—N1—C22	−122.88 (17)
C13—C8—C15—C16	−19.4 (3)	C8—C15—N1—C19	−83.58 (18)
N1—C15—C16—C17	−58.92 (19)	C16—C15—N1—C19	48.3 (2)
C8—C15—C16—C17	68.5 (2)	C5—C19—N1—C22	−65.7 (2)
N1—C15—C16—C20	175.44 (16)	C18—C19—N1—C22	170.53 (16)
C8—C15—C16—C20	−57.1 (2)	C5—C19—N1—C15	123.48 (16)
N1—C15—C16—C21	56.52 (19)	C18—C19—N1—C15	−0.2 (2)
