# Supplementary figures and images for: Crystal structure of 1-(2-chloro­acet­yl)-3,3-dimethyl-2,6-di-p-tolyl­piperidin-4-one
Source: Acta Crystallogr E Crystallogr Commun. 2015 Feb 13;71(Pt 3):o173–4. doi: 10.1107/S2056989015002613 (PMC4350729; doi:10.1107/S2056989015002613)

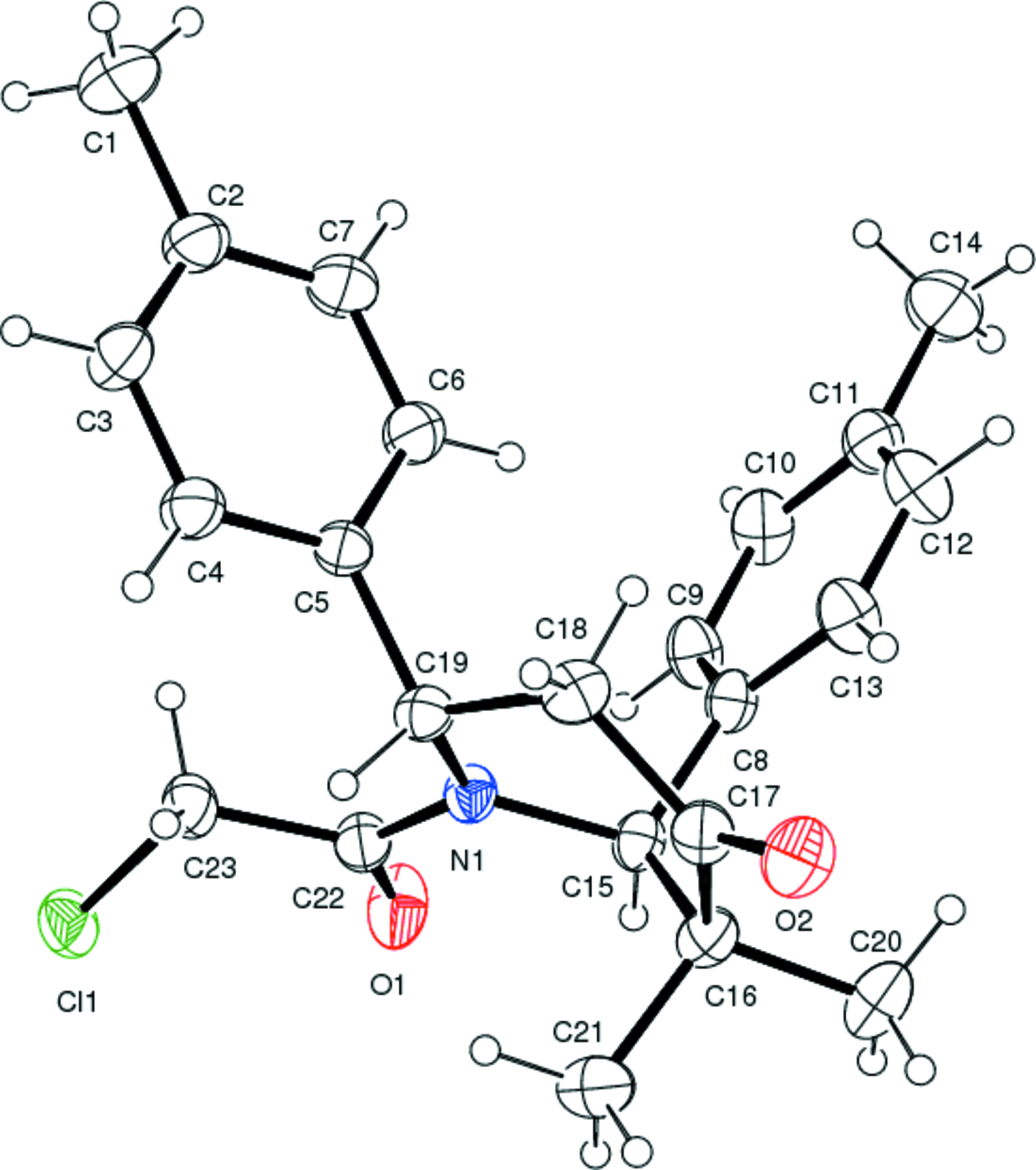

Supplement: Supplementary file 4 [file e-71-0o173-fig1.tif]
